# Investigation into the Antigenic Properties and Contributions to Growth in Blood of the Meningococcal Haemoglobin Receptors, HpuAB and HmbR

**DOI:** 10.1371/journal.pone.0133855

**Published:** 2015-07-24

**Authors:** Fadil A. Bidmos, Hannah Chan, Uta Praekelt, Isfahan Tauseef, Youssif M. Ali, Edward B. Kaczmarski, Ian Feavers, Christopher D. Bayliss

**Affiliations:** 1 Department of Genetics, University of Leicester, Leicester, United Kingdom; 2 National Institute for Biological Standards and Control, Potters Bar, United Kingdom; 3 Department of Infection, Inflammation and Immunity, University of Leicester, Leicester, United Kingdom; 4 Meningococcal Reference Unit, Public Health England, Manchester, United Kingdom; 5 Microbiology Department, Faculty of Pharmacy, Mansoura University, Mansoura, Egypt; Cornell University, UNITED STATES

## Abstract

Acquisition of iron from host complexes is mediated by four surface-located receptors of *Neisseria meningitidis*. The HmbR protein and heterodimeric HpuAB complex bind to haemoglobin whilst TbpBA and LbpBA bind iron-loaded transferrin and lactoferrin complexes, respectively. The haemoglobin receptors are unevenly distributed; disease-causing meningococcal isolates encode HmbR or both receptors while strains with only HpuAB are rarely-associated with disease. Both these receptors are subject to phase variation and 70–90% of disease isolates have one or both of these receptors in an ON expression state. The surface-expression, ubiquity and association with disease indicate that these receptors could be potential virulence factors and vaccine targets. To test for a requirement during disease, an *hmbR* deletion mutant was constructed in a strain (MC58) lacking HpuAB and in both a wild-type and TbpBA deletion background. The *hmbR* mutant exhibited an identical growth pattern to wild-type in whole blood from healthy human donors whereas growth of the *tbpBA* mutant was impaired. These results suggest that transferrin is the major source of iron for *N*. *meningitidis* during replication in healthy human blood. To examine immune responses, polyclonal antisera were raised against His-tagged purified-recombinant variants of HmbR, HpuA and HpuB in mice using monolipopolysaccharide as an adjuvant. Additionally, monoclonal antibodies were raised against outer membrane loops of HmbR presented on the surface of EspA, an *E*. *coli* fimbrial protein. All antisera exhibited specific reactivity in Western blots but HmbR and HpuA polyclonal sera were reactive against intact meningococcal cells. None of the sera exhibited bactericidal activity against iron-induced wild-type meningococci. These findings suggest that the HmbR protein is not required during the early stages of disease and that immune responses against these receptors may not be protective.

## Introduction

*Neisseria meningitidis* (Nm) is a frequent coloniser of the human oropharynx [1, 2] but is also a prolific pathogen. During a 15-year period (1996–2010), cyclic epidemics in the African meningitis belt affected 800,000 individuals with 30% having fatal outcomes or neurological sequelae (WHO, 2011). A significantly lower disease burden is reported in other parts of the world; however, localised epidemics occur with morbidity rates ranging from 0.28 cases per 100,000 in the United States to 2.4 cases per 100,000 in New Zealand [3]. Polysaccharide conjugate vaccines of different valences have been effective in preventing disease caused by strains of serogroups A, C, W and Y. Due to the potential for autoimmunity with a serogroup B polysaccharide-based vaccine [4], only epidemic-specific outer membrane vesicles were employed for controlling meningococcal disease of serogroup B aetiology [5–7]. Recently a novel recombinant protein-based vaccine, 4CMenB (also known as Bexsero) has been developed and licensed for prevention of MenB disease. This vaccine is predicted to provide protection against 70–90% of the meningococcal strains currently circulating in Europe [8, 9]. One aim of current meningococcal vaccine research is to extend coverage to additional strains by inclusion of additional vaccine antigens in a multi-component vaccine.

Nm possesses a myriad of nutrient scavenging and immune evasion systems. Iron is abundant in the human host but is rarely freely available with 30% and 66% of total body iron being complexed to ferritin or haemoglobin (Hb), respectively [10]. Iron-binding proteins such as lactoferrin [11], transferrin [12] and hepcidin [13] are involved in the sequestration of free extracellular iron, thereby creating an iron-limited environment in the human host. Some of these iron-binding proteins are implicated in nutritional immunity and a hypoferremic response during the early stages of infection [14]. Iron complexed to lactoferrin and transferrin serve as valuable iron sources for meningococci and are utilised via the bipartite LbpBA and TbpBA systems, respectively [15]. Each system is composed of a substrate-binding lipoprotein (LbpB and TbpB) and a transmembrane pore-forming protein (LbpA and TbpA). Expression of both systems is transcriptionally regulated by Fur and up-regulated in human whole blood [16], indicating the importance of these iron uptake systems to the meningococcus. Unsurprisingly, given the abundance of Hb in the human host, Nm can also acquire iron from Hb and Hb-complexes via two receptors, HpuAB and HmbR.

The HpuAB receptor is encoded by two co-transcribed genes, *hpuA* and *hpuB*, and is a bipartite receptor consisting of HpuA, a surface-exposed lipoprotein, and HpuB, a transmembrane protein [17]. HpuAB can bind both Hb and haemoglobin-haptoglobin (Hb-Hp) complexes [17] releasing haem which is then transported into the cell [18]. However, utilisation of free haem by gonococci is neither HpuAB nor TonB-dependent [19]. Expression of *hpuAB* is transcriptionally-regulated by Fur [17] and translationally-controlled by a polyG tract in the reading frame of *hpuA* [20]. HpuA cannot mediate Hb or Hb-Hp utilisation independently of HpuB but experimental data suggests that it contributes significantly to optimal binding of HpuAB to Hb and Hb-Hp. Conversely HpuB can mediate Hb utilisation in the absence of HpuA, albeit at levels lower than the functional HpuAB receptor [21–23]. The second Hb receptor, HmbR, is a TonB-dependent receptor of molecular mass ~89 kDa [24]. Expression of *hmbR* is phase-variable via a polyG tract within the reading frame [20, 25] and down-regulated under iron-replete conditions. Several isotypes of the Hb receptors exist with antigenic variation in HmbR being primarily determined by sequences of three putative surface-exposed loops [26–28].

The importance of these Hb receptors to meningococcal virulence has been demonstrated in an infant rat model where proliferation of a Δ*hmbR* mutant was attenuated [29] and in an accidental human passage that revealed a difference in the *hpuAB* expression status of the inoculum (*hpu*-OFF) and output (*hpu*-ON) populations [30]. These experimental data coupled with genetic studies that reported a bias for the presence and PV-ON status of one or both genes (*hpuAB* and *hmbR*) in disease isolates present strong indications that Hb-utilisation is crucial to meningococcal virulence [27, 31, 32]. While the constitutively-expressed meningococcal Tf receptor, TbpBA, has been established as an important virulence factor in Nm [33], the importance of the phase-variable Hb-acquisition systems, to survival and growth of the meningococcus in human whole blood is yet to be elucidated. Using an *ex vivo* human whole blood model, we examined the ability of phase-OFF variants of *hmbR* and *hpuAB* (or their equivalent i. e. mutants) to cause disease by proliferating in human blood. To ascertain the suitability of including these receptors in future vaccine preparations, we assessed the bactericidal property of serum antibodies generated in mice against purified recombinant HpuA, HpuB and HmbR.

## Materials and Methods

### Ethics statement

Studies with human blood samples were approved by the Leicester Research Ethics Committee. All volunteers provided written consent on a form approved by the ethics committee. Experiments with animals were approved by the NIBSC Ethics Committee and performed under home license PPL No. 80/2157) in accordance with the animal (scientific procedures) act 1986. Every effort was taken to minimize suffering. Anaesthesia without recovery was used to sacrifice animals.

### Bacterial strains

A list of Nm strains used in this study is presented in Table 1. Meningococcal carriage strains were obtained from a study conducted from November 2008 to June 2009 at the University of Nottingham [2]. Disease isolates, MC58 (B:P1.7,16–2:F1-5:CC-32), 8047 (B:P1.5–1,2–2:F3-6:ST-8) and H44/76 (B:P1.7,16:F3-3:CC-32) were isolated from meningococcal disease patients in the UK [34], US [35] and Norway [36], respectively. All strains were routinely grown in a humidified incubator at 37°C and 5% CO_2_ on Brain Heart Infusion (BHI), supplemented with Levinthal’s, or Mueller-Hinton (MH) media. Antibiotics were added to media, as required. *E*. *coli* strains DH5α and BL21 (DE3) were routinely grown in Luria agar or broth (LB) (Oxoid) supplemented with antibiotics, as required, at 37°C under aerobic conditions. For blue-white screening, an X-gal-IPTG mixture (10 mg/ml; Melford) was added to media at a working concentration of 10 μg/ml.

**Table 1:** List of *N*. *meningitidis* strains.

Strain	Source	Description
MC58	disease	B: P1.7,16–2: F1-5: CC-32 (*hpuAB*-, *hmbR*-ON)
MC58 ∆*hmbR*::*kan*	this study	*hmbR* deletion mutant of strain MC58
MC58 ∆*tbpBA*::*ery*	this study	*tbpBA* deletion mutant of strain MC58
MC58 ∆*hmbR*::*kan*∆*tbpBA*::*ery*	this study	*hmbR* and *tbpBA* deletion mutant of strain MC58
H44/76	disease	B: P1.7,16: F3-3: CC-32 (*hpuAB*-, *hmbR*-ON)
N66.1	carriage	B: P1.7–2,4: F1-5: CC-41/44 (*hpuAB*-, *hmbR*-ON)
N78.1	carriage	E: P1.21–7,16: F-*ND*: CC-1157 (*hpuAB*-OFF, *hmbR*-OFF)
N121.1	carriage	B: P1.22,14: F5-5: CC-213 (*hpuAB*-, *hmbR*-ON)
N123.1	carriage	NG: P1.21–7,16: F-*ND*: CC-1157 (*hpuAB*-ON, *hmbR*-ON)
N132.1	carriage	B: P1.22,14: F5-5: CC-213 (*hpuAB*-, *hmbR*-ON)
N176.1	carriage	B: P1.19,15: F5-1: CC-32 (*hpuAB*-ON, *hmbR*-ON)
N182.1	carriage	B: P1.12–6,13–4: F5-1: CC-32 (*hpuAB*-, *hmbR*-ON)
N193.1	carriage	NG: P1.18,25: F-*ND*: ST-845 (*hpuAB*-OFF, *hmbR*-ON)
8047	disease	B: P1.5–1,2–2: F3-6: ST-8 (*hpuAB*-ON, *hmbR*-OFF)
8047 ∆*hmbR*::*kan*	this study	*hmbR* deletion mutant of strain 8047
8047 ∆*hpuAB*::*kan*	this study	*hpuAB* deletion mutant of strain 8047
8047 ∆*tbpBA*::*ery*	this study	*tbpBA* deletion mutant of strain 8047
8047 ∆*hmbR*::*kan* ∆*tbpBA*::*ery*	this study	*hmbR* and *tbpBA* deletion mutant of strain 8047
8047 ∆*hpuAB*::*kan* ∆*tbpBA*::*ery*	this study	*hpuAB* and *tbpBA* deletion mutant of strain 8047
8047 ∆*hmbR*::*ery* ∆*hpuAB*::*kan*	[37]	*hmbR* and *hpuAB* deletion mutant of strain 8047
N88.1	carriage	Y: P1.21,16: F3-7: CC-174 (*hpuAB*-OFF, *hmbR*-)
N272.1	carriage	Y: P1.21,16: F3-7: CC-174 (*hpuAB*-ON, *hmbR*-)
N46.1	carriage	Y: P1.21,16: F3-7: CC-174 (*hpuAB*-ON, *hmbR*-)
N52.1	carriage	Y: P1.21,16: F3-7: CC-174 (*hpuAB*-ON, *hmbR*-)
N117.1	carriage	Y: P1.5–1,10–1: F1-3: CC-167 (*hpuAB*-ON, *hmbR*-)
N119.1	carriage	E: P1.5,2: F1-7: CC-60 (*hpuAB*-ON, *hmbR*-OFF)
N146.1	carriage	H: P1.21,16: F4-1: ST-*ND* (*hpuAB*-ON, *hmbR*-ON)
N199.1	carriage	B: P1.22,9: F5-5: CC-269 (*hpuAB*-ON, *hmbR*-OFF)
N222.1	carriage	Y: P1.5, 10–1: F4-1: CC-23 (*hpuAB*-ON, *hmbR*-)

### Induction of iron-regulated gene expression

Overnight liquid cultures, grown without iron-restriction, were diluted 20-fold into fresh media and incubated until the OD_600_ was ~0.5. Desferal (EMD Chemicals Inc.) was then added at a final concentration of 30 μM and the culture was incubated for a further 3 hours.

### Plasma and serum samples

Whole human blood was collected by qualified phlebotomists from healthy subjects in either sterile heparin-coated vacutainers for plasma or EDTA-coated vacutainers for sera (BD Vacutainer Systems, UK). For the preparation of sera, whole blood samples were coagulated on ice for 7 hours. A centrifugation step of 4000 rpm at 4°C for 10 minutes was used to separate serum from cellular material. Similarly, plasma was obtained from non-coagulated blood via centrifugation (4000 rpm at 4°C for 10 minutes).

### Construction of hmbR, hpuAB, and tbpBA deletion plasmids

Primers, HmbR-Nterm and dhmbR-rev (Table 2), were used to amplify a 542 bp region from the N-terminus of *hmbR* from strain MC58. Similarly, 676 bp from the C-terminus of *hmbR* was amplified with primers HmbR-Cterm and dhmbR-for (Table 2). These amplicons contained *BamH*I restriction sites, introduced via dhmbR-for and dhmbR-rev, which facilitated downstream ligation and sub-cloning steps. The ~1.3 kb fragment that resulted from ligation of both amplicons via the *BamH*I sites was cloned into the pGEM-T Easy plasmid vector, according to manufacturer’s instructions (Promega) to create pFAB-7. A kanamycin resistance cassette was excised from pUC4kan and inserted into the pFAB-7 via the *BamH*I site to give pFAB-9.

**Table 2:** Oligonucleotide primers for cloning.

Name	Sequence (5’-3’)	Purpose
HmbR-Nterm	CACCATGAAACCATTACAAATGCTCCC	Cloning of 5’ end of *hmbR*
dhmbR-rev	GCTGGATCCC AATCTGTATC	
HmbR-Cterm	TTAAAACTTCCATTCCAGCG	Cloning of 3’ end of *hmbR*
dhmbR-for	TGGGATCCATTCACGGTTACGC	
HpuA-Nterm	CACCGCCGAACCGCACGTCCCCG	Cloning of *hpuAB*
HpuB-Cterm	TTAGAACTTCGCTTCGATGG	
HA-Inv938	CTGATATCAGTGTCCCGGTAGCCG	Deletion of internal *hpuAB* fragment
HB-Inv1	CGGATATCGACTGGCGGTTTACCAAG	
HpuA-N-Pleic	TACTTCCAATCCATGGCCGAACCGCACGTCCCCGTGT	Cloning of HpuA into expression vector
HpuA-C-Pleic	TATCCACCTTTACTGTCAGGGAAACGCTTGGGCGAT	
HpuB-N-Pleic	TACTTCCAATCCATGGCGTTTCCCGCCTTTGCGGCAGA	Cloning of HpuB into expression vector
HpuB-C-Pleic	TATCCACCTTTACTGTCATTAGAACTTCGCTTCGATGGT	
HmbR-N-Pleic	TACTTCCAATCCATGGCAGATGAAGCTGCAACTGA	Cloning of HmbR into expression vector
HmbR-C-Pleic	TATCCACCTTTACTGTCATTAAAACTTCCATTCCAGCGAT	
HmbR loop2 F	GAAGGGGAAGGCAGTGGCGCGAATATCCGTGGTTCGGCACGCGGTATCCCTGATTCGTCCAAACACTTAGGCGCTGGTGATTTGCAA	Cloning of loop 2 of HmbR
HmbR loop2 R	GCCACTGCCTTCCCCTTCCACAGCATAGCCTCGGTTTCCCGCACTTTCGGTTTCATGACCGCGAGCAATTTTAGCATCTACAAG	
HmbR loop8 F	ACTGAGGAAAATGCTTACTACGGTATATGCAGCGACCCCTACAAAGAAAAATTAGGCGCTGGTGATTTGCAA	Cloning of loop 8 of HmbR
HmbR loop8 R	GTAAGCATTTTCCTCAGTACAGCCGGGAGTGCCGCTGGTGGTCAGCTTCTGAGCAATTTTAGCATCTACAAG	
HmbR loop11 F	GATGGCAAAGGCTTAGACCGCTACCGCGCCCCAGGCCGCAATTTAGGCGCTGGTGATTTGCAA	Cloning of loop 11 of HmbR
HmbR loop11 R	GTCTAAGCCTTTGCCATCGCGGTCGACCGCATTGGTGGTGCTAGCAATTTTAGCATCTACAAG	

For mutagenesis of *hpuAB*, the complete locus was amplified from strain 8047 with primers HpuA-Nterm and HpuB-Cterm and cloned in the pGEM-T Easy plasmid vector. An inverse PCR was performed on this plasmid with two primers, HA-Inv938 and HB-Inv1, with EcoRV sites on their 5’ ends. This product deletes 120 bp form the 3’ end of HpuA and 1,500 bp from the 5’ end of HpuB. PCR products were digested with EcoRV and then ligated with T4 DNA ligase prior to transformation into *E*. *coli* DH5α to create pGEMT-ΔhpuAB. A kanamycin cassette was inserted into the EcoRV site to create pGEMT-ΔhpuAB-Kan.

A *tbpBA* deletion construct (pIT-Δ*tbpBA*) was kindly provided by Isfahan Tauseef [37]. For the generation of double knockout strains, a different selectable marker for the deletion of *tbpBA* in the single knockout strains was required as both Δ*hmbR* and Δ*hpuAB* mutant strains were kanamycin resistant. The kanamycin selectable marker in pIT-Δ*tbpBA* was, therefore, replaced with an erythromycin resistance cassette flanked by one copy of the Neisserial DNA uptake sequence (DUS) on either end to yield pFAB-22.

### Natural transformation of Nm

A modification of the transformation protocol described in [38] was employed in the present study. Briefly, a 1:10 dilution of cells grown overnight in supplemented BHI medium was incubated for one hour with shaking at 37°C. 200 μl of the culture was added to supplemented BHI agar in 24-well plates. The culture was incubated for 5 hours at 37°C, 5% CO_2_ before 1.5 μg of the *Nde*I-linearised plasmid was added. The transformation mixture was incubated overnight; subsequently, cells were harvested and plated on selective BHI agar plates. Colonies present on selective plates after 24 hours were confirmed as Nm mutants using specific PCR (amplification of short regions of the *ctrA* and *crgA* genes and of the junction between the antibiotic cassette and mutated gene) and disc diffusion assays.

### Disc diffusion assay

Approximately 10^9^ Nm cells were seeded on iron-chelated Mueller-Hinton (MH) agar. Sterile filter discs (5 mm in diameter) infused with an iron source [500 μg of human holo-transferrin (Sigma-Aldrich), 100 μg of ferrous-stabilised human haemoglobin A0 (Sigma-Aldrich) or 100 mM FeCl_3_] were carefully placed on different sections of the plate. Alternatively, 5 μl of the exogenous iron sources were dropped on different sections of the plate. Sterile PBS buffer was also included as a negative control. Plates were incubated overnight for 24 to 48 hours until rings of growth were clearly visible around the iron sources.

### Generation of polyclonal antisera in mice

For expression of recombinant HpuA, HpuB and HmbR proteins from strains N88.1, 8047 and MC58 respectively, amplicons were generated with specific primers (Table 2) and cloned into a pLEICS-03 vector (PROTEX laboratory, University of Leicester, UK). For *hpuA*, the N-terminal primer was located immediately downstream of the repeat tract whilst for the other genes the primer binding site started from the initiation codon. The *hmbR* gene was amplified from isolates with an ON number of repeats and the constructs was confirmed to have retained an in-frame repeat tract. Expression of recombinant protein was induced in transformed *E*. *coli* BL21 (DE3) cultures at OD_600_ of ~ 0.5 with IPTG at a final concentration of 1 mM.

Recombinant HpuA was purified from the soluble fraction of BL21 cell lysates using a Ni-Sepfast gravity column, as per manufacturer’s instructions (Flowgen Bioscience). Inclusion bodies containing recombinant HpuB and HmbR were purified by cell lysis and washing in 50 mM TrisHCl pH 8. The inclusion bodies were then treated with solubilisation buffer (20 mM Tris.Cl pH 8.0, 6M guanidine HCl, 1mM EDTA) and applied in a drop-wise manner to nine volumes of refolding buffer (20 mM Tris.Cl pH 8.0, 5% v/v LDAO, 250mM NaCl, 50μM Hemin) on a magnetic stirrer. The solubilised protein was allowed to refold for 4 hours at room temperature and the resulting haem-bound proteins dialysed overnight at 4°C against dialysis buffer (20 mM Tris.Cl pH 8.0, 0.1% v/v LDAO). The identity of recombinant proteins was confirmed using mass spectrometry (Protein and Nucleic Acid Chemistry Laboratory, University of Leicester, UK).

Six groups of 10 female BALB/c mice (6–7 weeks old) were subcutaneously injected with 200 μl of antigen preparations (20 μg antigen + 10 μg monophosphoryl lipid A) as follows:- r8047-HmbR (group 1); rMC58-HmbR (group 2); r8047-HpuA (group 3); rN88-HpuA (group 4); r8047-HpuB (group 5). The sixth group received no antigen (MPL only). Boosters, containing 200 μl of the antigen preparations as above, were administered at weeks 3 and 5. Terminal bleeds were obtained at week 7.

### Production of anti-HmbR monoclonal antibodies

Epitopes corresponding to predicted surface-exposed loops 2 (HmbR_193-229_), 8 (HmbR_550-577_) and 11 (HmbR_739-750_) of HmbR from strain MC58 were amplified with specific primers (Table 2) and used to replace an immunogenic loop of the *E*. *coli* EspA protein. Three mice were immunised with each of the antigen preparations while a fourth mouse received a combination of all three antigens. Test bleeds were first screened for presence of antibodies by ELISA and probing of Western blots using recombinant EspA-HmbR and meningococcal lysates as target antigens. Monoclonal antibodies were produced from immortalised spleen cells of mice obtained after 7 weeks as described by Praekelt *et al*., [39]. Briefly, spleen cell-NS0 hybridomas were generated and selected following screening of secreted antibodies by ELISA against recombinant EspA-HmbR, purified His-tagged HmbR and meningococcal cell lysates. Positive hybridomas were subsequently sub-cloned twice. Supernatants from these hybridomas were stored at -20°C.

### Western and colony immunoblotting

Cell lysates and protein samples were analysed on 10% SDS-PAGE gels. Electrophoresis was performed at a constant voltage of 80 V for 3 hours. Electrophoresed samples were transferred from SDS-PAGE gels onto pre-activated polyvinylidene fluoride (PVDF) membranes (Merck Millipore) using ice-cold Towbin transfer buffer (25 mM Tris, 192 mM glycine, 20% methanol). Transfer was done for 1 hour at constant ampere of 150 Amp before membranes were blocked overnight at 4°C with blocking buffer (PBS, 0.5% Tween-20, 5% Milk). Following blocking, membranes were incubated with an appropriate concentration of primary antibodies in blocking buffer for 1 hour at room temperature with gentle shaking. Membranes were washed thrice with wash buffer (PBS, 0.5% Tween-20) and subsequently incubated in a 1:2000 dilution of anti-mouse horseradish peroxidase (HRP), also prepared in blocking buffer, for 1 hour at room temperature. Membranes were washed thrice before signals were developed with an EZ-ECL Chemiluminescence kit for HRP (Geneflow). Blots were quantified with ImageJ software.

For assessment of protein surface expression with colony immunoblotting, ~ 10^9^ Nm cells were spotted on BHI agar plates supplemented with 10% Levinthal’s and 65 μM desferal. After an overnight incubation, growth was transferred onto blotting membranes for 10 minutes. Membranes were subsequently blocked with blocking buffer for 1 hour at room temperature. Excess cellular material was gently removed from membranes before incubation with primary antibodies (1:100 in blocking buffer) for 2 hours at room temperature. Unbound primary antibodies were removed by washing membranes thrice with wash buffer. The wash step was immediately followed by incubation with a 1:2000 dilution of an anti-mouse IgG HRP or alkaline phosphatase conjugate antibody. After 1 hour, membranes were washed and signals developed using either an EZ-ECL kit (GeneFlow) or BCIP/NBT substrate solution (Perkin Elmer).

### Flow cytometry

Analysis of protein surface expression by flow cytometry was performed as described previously [40]. Briefly, 150 μl of cells, cultured in iron-replete or iron-restricted conditions, were pelleted by centrifugation at 8,000 rpm for 1 minute in a microcentrifuge and washed twice with assay buffer (50 mM Tris.Cl pH 7.5, 150 mM NaCl, 5 mM CaCl_2_, 0.05% Tween-20). Cells were subsequently incubated with a 1:20 dilution of antigen-specific antibodies prepared in 150 μl assay buffer at room temperature for 1 hour after which unbound antibodies were removed in three wash cycles. This was followed by addition of 20 μg/ml of the secondary antibody, Alexa Fluor 488 Goat Anti-Mouse IgG (H+L) (Life Technologies), and incubation for 1 hour at room temperature. Three wash cycles were performed before cells were inactivated in 1 ml of fixer solution (0.05% formalin in PBS). Samples were analysed on a BD FACSCalibur system.

### Human whole blood growth assay

The protocol for the whole blood assay was adapted from previous studies [41, 42]. Blood samples were collected from mannose-binding lectin (MBL)-deficient or normal donors. MBL deficiency has been linked with an increased susceptibility to meningococcal disease [43] and an active MBL pathway can accelerate opsonophagocytosis of Nm by macrophages [44]. Thus, whole blood samples from these volunteers were expected to be less inhibitory of meningococcal survival and growth. A 1:20 dilution of an overnight Nm culture was used to inoculate MH broth supplemented with 0.25% glucose and 20 μM CMP-N-acetylneuraminic acid sodium salt (CMP-NANA) for exogenous sialylation of Nm LOS. Approximately 10^6^ cells were taken from mid-logarithmic phase cultures and used to inoculate 500 μl of freshly-collected heparinised human blood (0.01 U of heparin/μl of blood) in 24-well plates. Blood cultures were incubated at 37°C, 5% CO_2_ for 8 hours. CFU counts of Nm cells pre-inoculation into blood, immediately after inoculation (T0) and at hourly intervals were taken. Doubling times were estimated using Doubling Time software.

### Human serum bactericidal assay (hSBA)

A modified protocol was used to assess serum sensitivity of meningococcal strains and the bactericidal activity of monoclonal and polyclonal antibodies. Liquid cultures were grown until an OD600 of ~0.5 was reached. Where iron restriction conditions were required, 30 μM desferal was added and grown for a further three hours to induce expression of iron-regulated genes. Cultures were subsequently diluted to an OD_600_ of 0.1 in PBSB (PBS, 0.15 mM CaCl_2_, 0.5 mM MgCl_2_). For the assay, the following components were transferred to specified wells of a 96-well plate:- 2-fold serial dilutions of antibodies (note that the complement in polyclonal antisera was first inactivated by incubation of sera at 56°C); 10 μl of a 1:2500 dilution of the bacterial cell suspension (expected to contain ~10^4^ CFU); the exogenous complement source (i.e. pooled human serum) and assay buffer to a total volume of 50 μl. Three control assays were setup on each plate to assess: antibody-independent serum sensitivity to exogenous complement (cells plus complement only), complement-independent killing by antibodies (cells plus antibodies and heat-inactivated complement) and the fitness of cells used in the assay (duplicate wells containing cells plus heat-inactivated complement, one of which was plated prior to incubation to obtain a T_0_ count). Plates were subsequently incubated at 37°C for 1 hour. Samples from all assay wells were plated at the end of the experiment to provide T_60_ counts.

## Results

### Construction of meningococcal strains lacking haemoglobin and transferrin receptors

**Fig 1:**
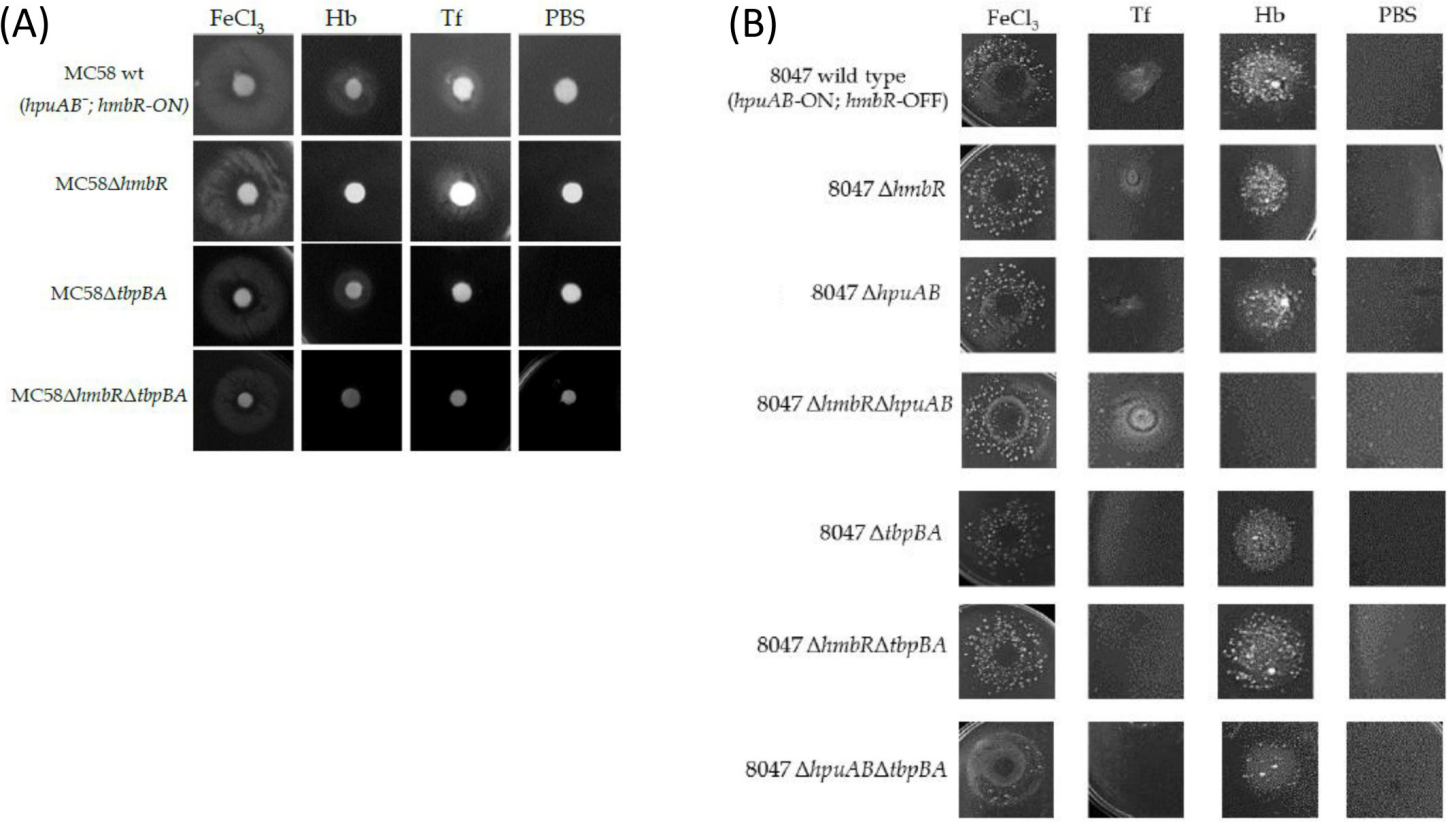
Utilization of Hb and Tf by wild-type and mutant *N*. *meningitidis* strains. Desferal (40 μg/ml) was added to molten MH agar to chelate available iron in the agar. A suspension of 10^8^ cells from an overnight culture was spread onto each plate before sterile filter discs infused with either 100 μg of Hb or 500 μg of Tf were placed on the agar. Plates were incubated at 37°C, 5% CO_2_ for 24 hours. Discs infused with FeCl_3_ and PBS were used as positive and negative controls. Panel (A) MC58; panel (B) 8047.

In order to investigate the contributions of the haemoglobin binding proteins of *N*. *meningitidis* to the ability of meningococci to cause invasive disease, parts of the *hmbR* and *hpuAB* loci were deleted and replaced by antibiotic cassettes. Mutations were constructed in a strain with only the HmbR receptor, strain MC58, and one, strain 8047, with both receptors (i.e. HmbR and HpuAB). The *tbpBA* genes were also inactivated by a similar method in both wild-type (wt) and *hmbR* mutant backgrounds. The repeat tracts of the Hb receptors in the wt strains and *tbpBA* mutants were sequenced, HmbR was in the ON state in the MC58 strain-background but OFF in strain 8047 wherein HpuAB was in an ON state. Growth assays showed similar doubling times for the wt and mutant strains in MH broth (data not shown). Phenotypic characterisation of wt and mutant MC58 strains was then performed using a disc diffusion assay. The MC58Δ*hmbR* utilized Tf but not Hb whilst the converse was observed for MC58Δ*tbpBA* (Fig 1A). The MC58 double mutant was unable to utilize Tf or Hb whilst the wt strain utilised both (Fig 1A). Similarly, only the 8047Δ*hmbR*Δ*hpuAB* mutant was unable to utilise Hb while all 8047Δ*tbpBA* mutants failed to grow when Tf was the sole iron source (Fig 1B). The ability of Δ*hpuAB* mutants to utilise Hb could be due to the presence of small numbers of *hmbR*-ON phase variants in the inoculum. All strains utilized free iron provided in the form of 0.1 M FeCl_3_ (Fig 1). The *hemO* gene is located upstream of the *hmbR* gene and HemO is required for utilisation of haem derived from Hb [45]. All mutant strains are assumed to have retained HemO function as indicated by the ability of 8047Δ*hmbR* to grow on Hb presumably through the combined actions of HpuAB and HemO.

### Absence of HmbR does not impair growth of meningococci in human whole blood

**Fig 2:**
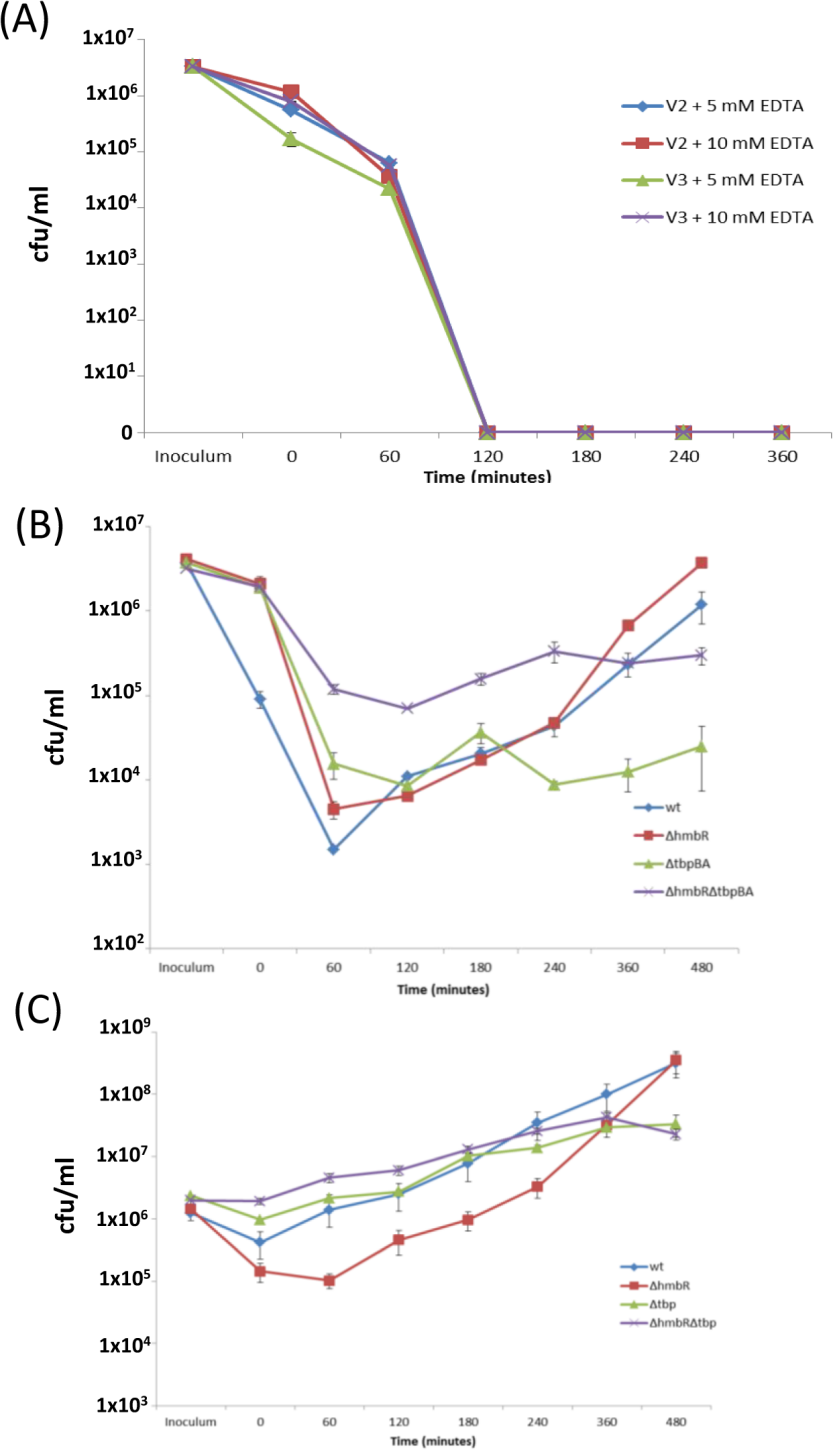
Growth of Hb and Tf receptor mutants of *N*. *meningitidis* in whole human blood. Bacterial strains (8047, MC58 and mutants thereof) were grown to mid-log (OD600 = ~ 0.6) in MHB supplemented with 20 μM CMP-NANA and then added to freshly-collected human blood. Cultures were incubated at 37°C, 5% CO_2_ for 4 hours. Samples, collected from the inoculum and at 0, 60, 120, 180 and 240 minutes from blood cultures, were plated in duplicate onto supplemented BHI agar. The Fig shows a representative experiment. Each strain and mutant was tested in triplicate and intra-assay variation was measured by calculating the standard error of the mean of the CFU counts obtained at each timepoint. Blood samples were collected from two healthy MBL-deficient volunteers (V1 and V2) and an uncharacterised volunteer (V3). (A) strain 8047 (wt) in blood from V2 and V3, which was treated with either 5 mM or 10 mM EDTA; (B) wild-type and mutants of strain MC58 in untreated blood from V1; (C) wild-type and mutants of strain MC58 in untreated blood from V2.

Sensitivity of Nm to the bactericidal activity of whole blood has been shown previously [41, 42]. Use of donors (V1 and V2) deficient in the mannose-binding ligand (MBL, a known activator of complement-mediated killing of meningococci) and growth of strains with an exogenous sialylation source prior to inoculation into the blood was pursued in order to increase bacterial survival. Despite these precautions, both *wt* and mutant 8047 strains were not recoverable from blood after 60 minutes (data not shown). Addition of either 5 mM or 10 mM EDTA to inactivate the complement system in either an MBL-deficient (V1) or normal donor (V3), in addition to exogenous sialylation, did not result in a reduction of the bactericidal activity of whole blood to strain 8047 (Fig 2A). The susceptibility of strain 8047 to 20% human serum (individually tested against serum from two separate donors) but not 5% serum suggests that killing of strain 8047 in whole blood was complement-mediated (data not shown). Hence, the impact of the absence of *hpuAB* on growth in the human whole blood model was unattainable in this study.

Decreases in CFU counts were observed immediately after inoculation of whole blood with strain MC58 but with a much higher level of killing in blood from the V2 as compared to the V1 donor (Fig 2B and 2C). For both donors, the Δ*tbpBA* mutants were less sensitive to the bactericidal activity of the blood than the wt or HmbR knock-out mutant. Following these initial reductions in CFU, both MC58 wt and MC58Δ*hmbR* grew exponentially from T_60_ to T_480_ in blood from both donors with average doubling times of 49 and 36 minutes, respectively. Both Δ*tbpBA* mutants exhibited a lower growth rate in both V1 and V2 blood with average doubling times of 80 (MC58Δ*tbpBA*) and 93 (MC58Δ*hmbR*Δ*tbpBA*) minutes. Growth of the single and double Δ*tbpBA* knockout strains in human whole blood was significantly different to those obtained in MH broth, as doubling times of 45 (MC58Δ*tbpBA*) and 48 (MC58Δ*hmbR*Δ*tbpBA*) minutes were recorded (one-tailed paired t-test; p = 0.04) whereas the *wt* and Δ*hmbR* strains exhibited similar growth rates in blood and MH broth (p = 0.29).

### Anti-HpuA and anti-HmbR antibodies recognise surface-exposed variant-specific epitopes

The antigenic diversities of *hpuAB* and *hmbR* are mainly due to the presence of variable regions (VR), which encode surface-exposed immunodominant loops [28, 31]. The degree of cross-reactivity between meningococcal strains expressing different antigenic types of these receptors was examined using polyclonal antisera raised against recombinant proteins purified from *E*. *coli* extracts. Mouse sera containing anti-r8047-HpuA, anti-rN88-HpuA and anti-r8047-HpuB antibodies were used to probe iron-starved cells of strain 8047, 8047 Δ*hpuAB* mutant (8047Δ*hpuAB*), N88.1 (*hpuAB*-OFF) and N272.1 (*hpuAB*-ON) by Western blotting, flow cytometry and colony immunoblotting assays. The N88.1 and N272.1 isolates were sequentially isolated from the same healthy volunteer [2] and were shown to be *hpuAB* phase variants of the same strain (Y:P1.21,16:F3-7:CC-174); [46]).

**Fig 3:**
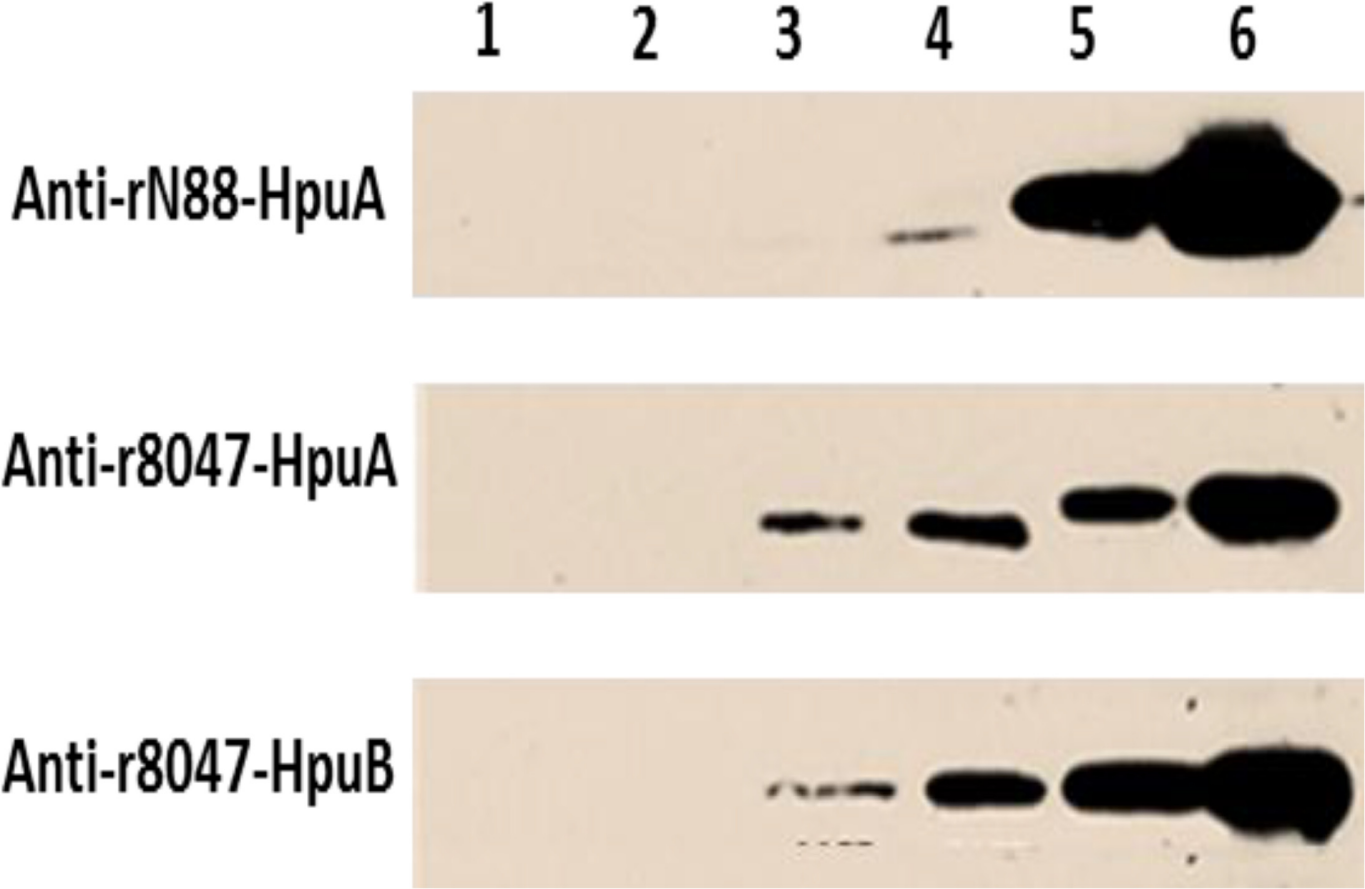
Reactivity of anti-rHpuA and anti-rHpuB antisera with HpuAB in meningococcal lysates. Meningococcal cells of strain 8047, an isogenic Δhpu mutant and two phase variants of a carriage strain (N88.1, hpu-OFF; and N272.1, hpu-ON) were grown to mid-log (OD600 = ~0.5) before 30 μM of desferal was added to produce iron-limited conditions. Cultures were also grown concurrently in iron-replete conditions. All cultures were incubated for two hours before heat-inactivation at 56°C overnight. Lysates were prepared using an equal number of OD units and subject to SDS-PAGE electrophoresis and Western blotting. Blots were then probed with 1:500 dilutions of mouse polyclonal sera followed by a 1:2000 dilution of an anti-mouse IgG HRP-conjugate:- upper panel, anti-rN88-HpuA; middle panel, anti-r8047-HpuA; lower panel anti-r8047-HpuB. Lysates were from:- strain N88.1, induced (lane 1); 8047ΔhpuAB, induced (lane 2); wild-type 8047, uninduced (lane 3); wild-type 8047, induced (lane 4); N272.1 uninduced (lane 5); and N272.1 induced (lane 6).

**Fig 4:**
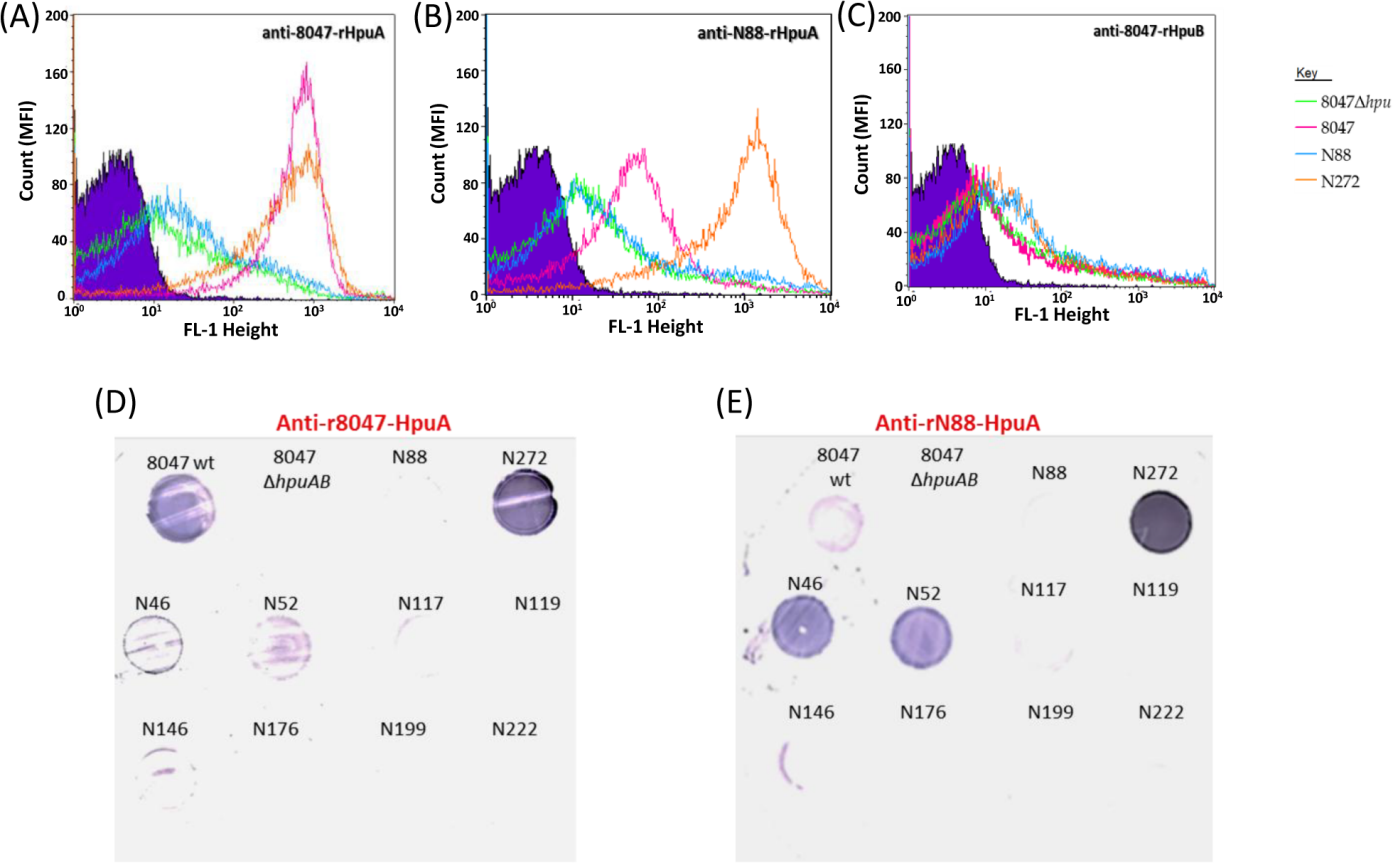
Evaluation of HpuA and HpuB surface expression by flow cytometry and immunoblotting. Cultures of meningococcal strains 8047ΔhpuAB, 8047 wild-type, N88.1 and N27.12 were subjected to growth in iron-limited conditions (see Fig 3). Formalin-fixed cells were assessed for surface expression of HpuA or HpuB in a FACS assay (panels A-C) by incubation with a 1:20 dilution of a polyclonal mice antiserum followed by a 1:100 dilution of a secondary antibody (Alexa Fluor anti-Mouse IgG) and detection of fluorescence in a flow cytometer. A total of 25,000 events were analysed for each assay. Primary antisera were:- (A) anti-r8047-HpuA; (B) anti-rN88-HpuA; (C) anti-r8047-HpuB. Strain are as indicated in the key. Immunoblotting (panels D-E) was performed using meningococcal cells grown on supplemented BHI agar containing 65 μM desferal and incubated at 37°C, 5% CO_2_. Cells were transferred to nitrocellulose filters and probed with a 1:250 dilution of either an anti-r8047-HpuA (D) or an anti-rN88-HpuA (E) followed by a 1:2000 dilution of an anti-Mouse IgG AP-conjugate.

Both anti-rHpuA mouse antisera bound to a protein of the expected size (~40-kDa) in Western blots of meningococcal lysates of strain 8047 and CC174 *hpuAB*-ON variant (N272.1) but not the 8047Δ*hpuAB* mutant or the CC174 *hpuAB*-OFF variant (N88.1), indicating specific reactivity with HpuA (Fig 3). Surface expression of HpuA in the 8047 and N272.1 strains was demonstrated by detection of high reactivity to formalin-fixed cells in the FACs assays (Fig 4A and 4B). No difference was observed, by Western blotting or FACs, in binding of anti-r8047-HpuA antibodies to strains N272.1 and 8047 whereas the anti-rN88-HpuA sera exhibited a major reduction in reactivity with 8047-HpuA as compared to N88.1-HpuA. This suggests that there are either differences in the level of HpuA expression, with a lower level of expression in strain 8047 as compared to the CC174 strain, or differences in the amounts of variant specific antibodies in each serum (i.e. more variant-specific antibodies in the N88 versus the 8047 sera). Further testing of the reactivity of anti-HpuA antisera to a panel of carriage strains in colony immunoblots showed specific reactivity of anti-rN88-HpuA to only the cognate CC-174 strains, N46 and N52 (Fig 4E). These CC-174 isolates were isolated from the same hall of residence and represent a cluster of genotypically identical strains that are thought to be a product of clonal expansion within the hall of residence [2]. A very weak interaction between anti-r8047-HpuA antibodies and the CC-174 strains was observed but no reactivity with other strains in the panel could be discerned (Fig 4D).

No discernible reactivity by anti-r8047-HpuB to surface-exposed HpuB regions in any of the four strains analysed was observed (Fig 4C) whereas the Western blots showed clear and specific reactivity with HpuB in whole cell lysates (Fig 3). The absence of reactivity between anti-rHpuB antibodies and surface-expressed HpuB could be due to a lack of accessibility due to blocking by HpuA, conformational changes to HpuB occurring during formation of a receptor complex with HpuA [21] or improper folding of HpuB during preparation of recombinant protein.

**Fig 5:**
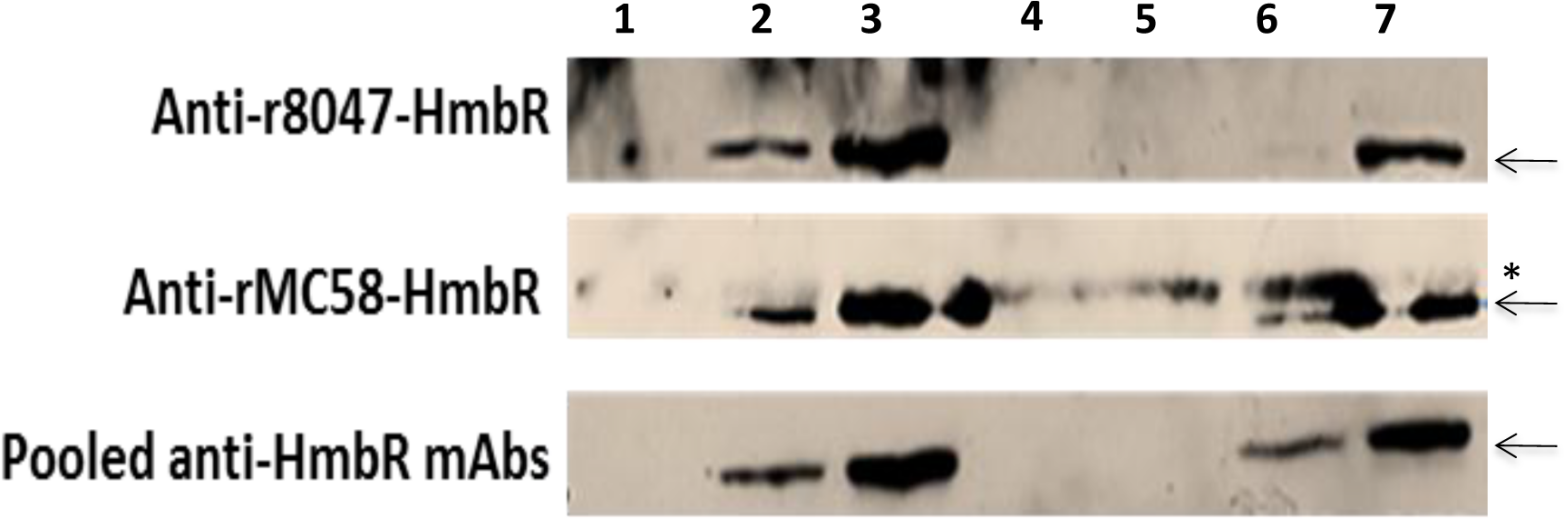
Reactivity of anti-HmbR monoclonal and polyclonal antisera with HmbR in meningococcal lysates. Meningococcal lysates were prepared from cells grown in iron-limited (induced) or iron-replete (uninduced) conditions as described for Fig 3. Equal amounts of cells were analysed by probing Western blots with a 1:500 dilution of a primary antiserum followed by a 1:2000 dilution of an anti-Mouse IgG HRP conjugate. Upper panel, anti-r8047-HmbR; middle panel, anti-rMC58-HmbR; lower panel, pooled mAbs raised against two surface exposed epitopes of the MC58 HmbR protein. Lysates were:- induced strain MC58ΔhmbR (lane 1); uninduced wild-type MC58 (lane 2); induced wild-type MC58 (lane 3); uninduced wild-type 8047 (lane 4); induced wild-type 8047 (lane 5); uninduced wildtype H44/76 (lane 6); induced wildtype H44/76 (lane 7). Arrows, HmbR; asterisk, non-specific reactive protein.

**Fig 6:**
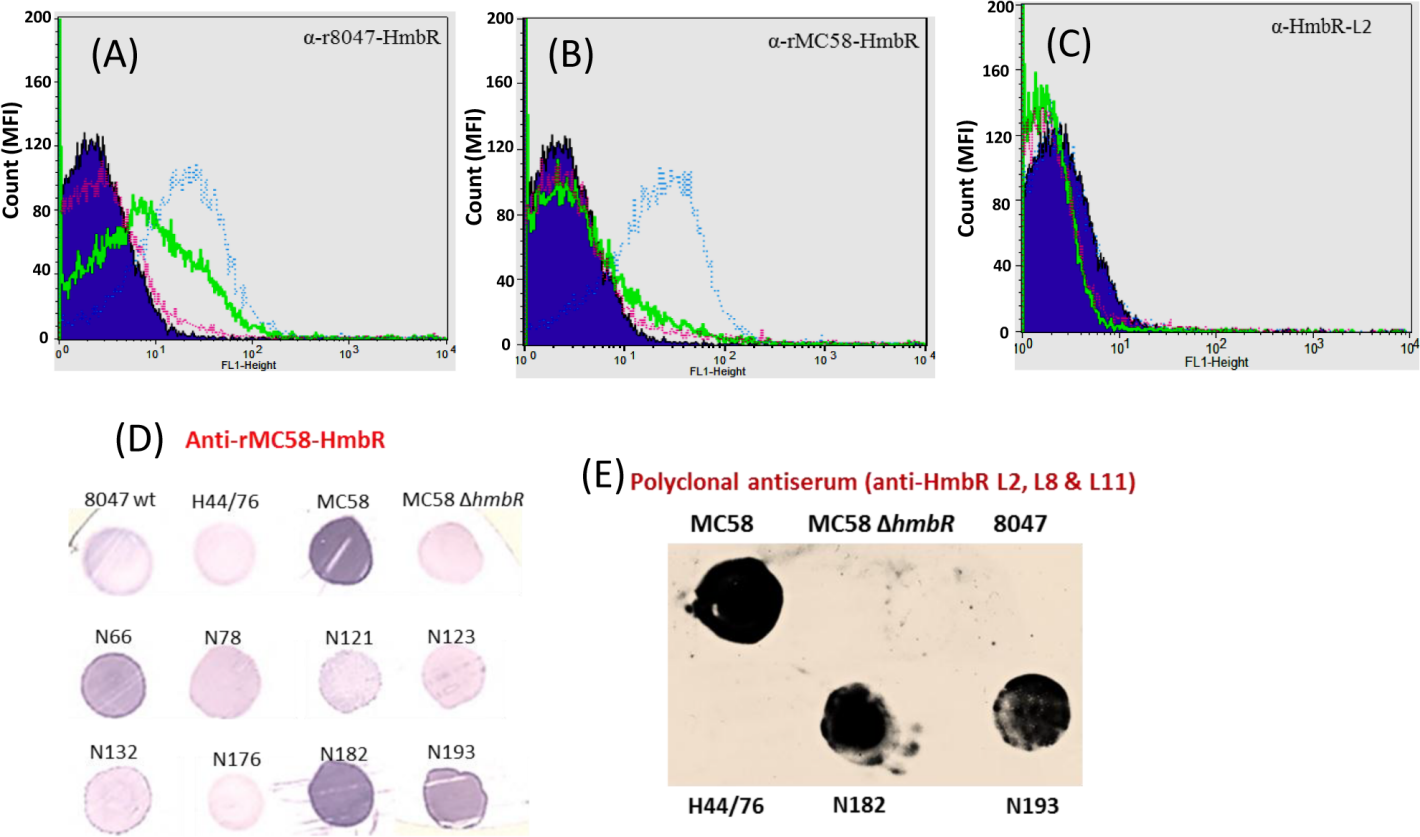
Evaluation of HmbR surface expression by flow cytometry and colony immunoblotting. Cultures of meningococcal strains were subjected to growth in iron-limited or iron-replete conditions as described in Fig 3. Formalin-fixed cells were subsequently assessed for HmbR surface expression in a FACS assay using a 1:20 dilution of a primary antibody followed by a 1:100 dilution of a secondary antibody (Alexa Fluor anti-Mouse IgG) and detection of fluorescence in a flow cytometer. Primary antibodies were:- (A) a polyclonal anti-r8047-HmbR antiserum; (B) a polyclonal anti-rMC58-HmbR antiserum; and (C) L11, an anti-HmbR monoclonal antibody directed against surface-exposed loop 11. Strains were:- MC58ΔhmbR, purple line; MC58, blue line; 8047, green line; and H44/76, red line. Colony immunoblots were also performed using meningococcal cells grown overnight on supplemented BHI agar with 65 μM desferal at 37°C, 5% CO_2_. Cells were transferred to nitrocellulose filters and probed with a 1:100 dilution of either a polyclonal anti-HmbR antiserum (D) or a polyclonal serum raised against carrier proteins containing surface-exposed loops L2, L8 and L11 (E). The secondary antibody was an anti-mouse IgG HRP-conjugate, which was used at a 1:2000 dilution.

The reactivity of anti-r8047-HmbR and anti-rMC58-HmbR polyclonal antisera to Nm was assessed by probing lysates (Fig 5) and formalin-fixed cells (Fig 6) of strains 8047 (*hmbR*-OFF), H44/76 and MC58 (both *hmbR*-ON but antigenically dissimilar) or an MC58Δ*hmbR*. Both polyclonal antisera bound to a protein of ~90-kDa in iron-induced MC58 and H44/76 cells but not to MC58Δ*hmbR*, 8047 or uninduced cells. The anti-rMC58-HmbR serum showed cross-reactivity with a protein of a slightly larger size accounting for the reactivity with strain 8047, which is HmbR OFF. Consistent results were obtained using colony immunoblots (Fig 6D and 6E). In flow cytometry assays (Fig 6A and 6B), however, the polyclonal sera only showed reactivity to induced cells of strain MC58 and not to H44/76, indicating that epitopes detected in the Western and colony-immuno blots with this strain were not surface-exposed. A low degree of reactivity to iron-starved 8047 cells was observed with the homologous anti-r8047-HmbR antibody, possibly due to the presence of small numbers of *hmbR*-ON phase variants in the population (Fig 6A). In addition to tight binding to MC58 cells, the anti-rMC58-HmbR serum was reactive with carriage strains belonging to three different clonal complexes (N66.1, CC41/44; N182.1, CC32; and N193.1, ST-845). Interestingly, no reactivity to strain N176.1, which is antigenically homologous to MC58 with respect to *hmbR*, was found (Fig 6D). Sequence analysis of the N176.1-*hmbR* gene revealed a premature stop codon, which was responsible for the non-expression of *hmbR* and the consequent lack of a discernible interaction between the anti-rMC58-HmbR serum and N176.1 cells.

### Generation of monoclonal HmbR antibodies

Expression of surface-exposed loops in a heterologous carrier protein was explored as an alternative strategy for generation of bactericidal antibodies against HmbR. Using the structural predictions contained in Evans *et al*. [28] for HmbR from strain MC58, the three largest surface-exposed loops (L2, L8 and L11) were selected with L2 also being one of the loops exhibiting the highest strain-to-strain variability and, therefore, presumed to be a major target of antibody responses. These three surface loops of HmbR (see Materials and Methods) were cloned from strain MC58 and inserted into EspA, an *E*. *coli* fimbrial protein. Polyclonal antisera were raised in mice against mixtures of these recombinant proteins and three hybridomas producing monoclonal antibodies specific for the *hmbR* loops were successfully cloned from this serum. Two of the mAbs targeted loop 11 of *hmbR* and the third recognised loop 2. All three mAbs bound to HmbR in meningococcal cell lysates of strain MC58 (Fig 5) but showed no reactivity to surface-expressed HmbR by flow cytometry or colony immunoblotting (Fig 6C and data not shown). Conversely, a polyclonal antiserum containing antibodies to all three HmbR-EspA fusion proteins was highly reactive with surface-expressed HmbR in colony immunoblots and specifically recognised HmbR in cell lysates (Figs 5 and 6). The poor binding of mAbs to surface-expressed HmbR may be due to a combination of low concentrations of antibodies and small amounts of surface-expressed HmbR.

### Anti-rHpuA and anti-rHmbR antibodies are not bactericidal

An assessment of the bactericidal properties of antibodies targeting surface-expressed HpuA and HmbR was performed with iron-starved Nm cells as both proteins are induced under low iron conditions. This approach was previously employed during testing of the vaccine candidacy of TbpA and TbpB [47–49]. Active human complement was derived from volunteers whose sera had low levels of bactericidal activity against Nm [40].

**Fig 7:**
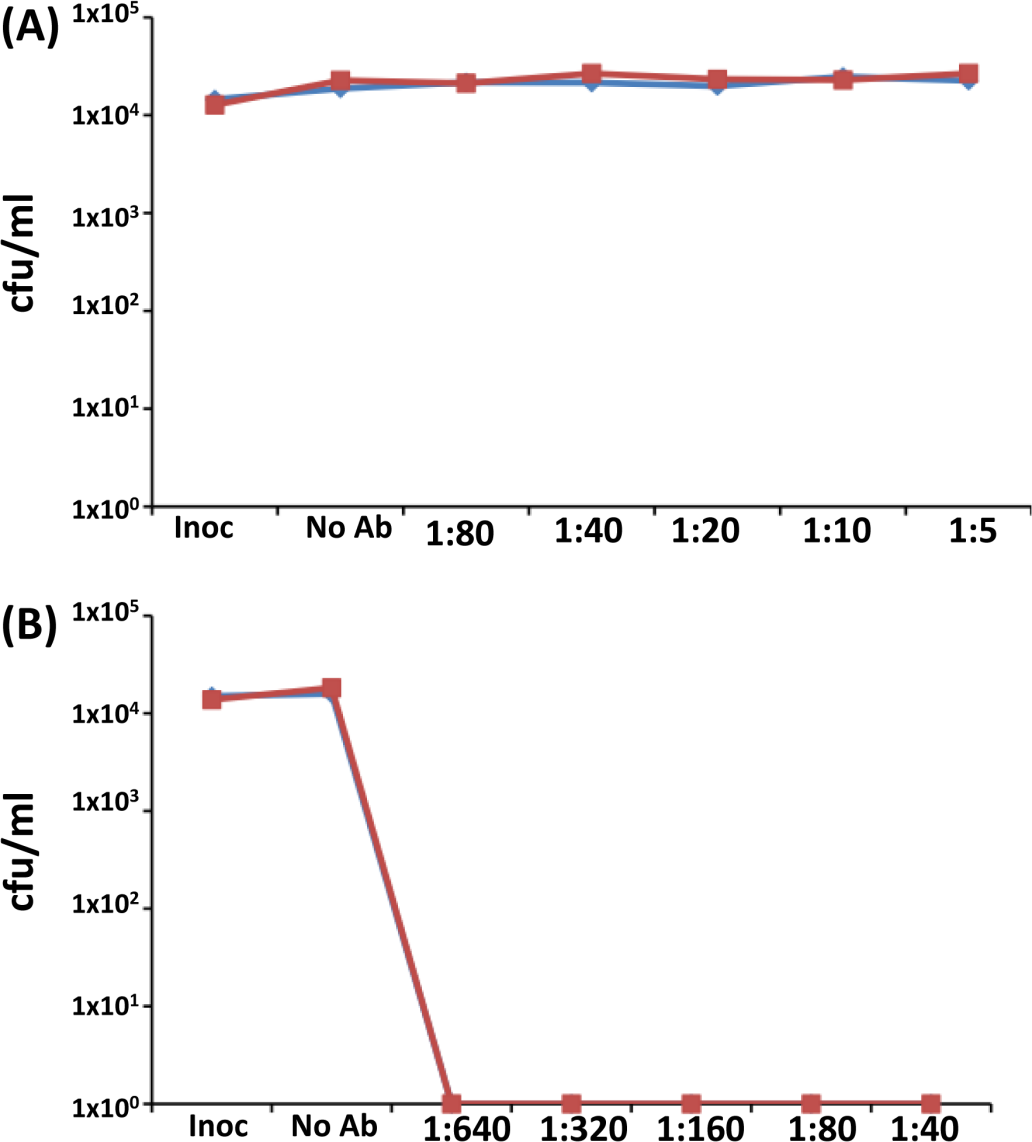
Testing the serum bactericidal activity of anti-rHpuA antisera. Meningococcal cell suspensions were prepared following growth under iron-restricted conditions. An aliquot of 10^4^ cfu was mixed with pooled human complement at a final concentration of 5% and dilutions (as indicated on the x-axis) of either a polyclonal anti-rHpuA antiserum (A) or anti-PorA monoclonal antibody P1.2 (B). Assays were incubated at 37^°^C for 60 minutes and then assessed for the numbers of surviving cells (cfu/ml) by plating 10μl of aliquots on supplmented BHI agar. Inoc, cell count after 0 minutes; No Ab, cell count after 60 minutes incubation in serum alone. Graphs show mean values for two independent experiments. Red line, 8047; blue line, 8047ΔhpuAB.

**Fig 8:**
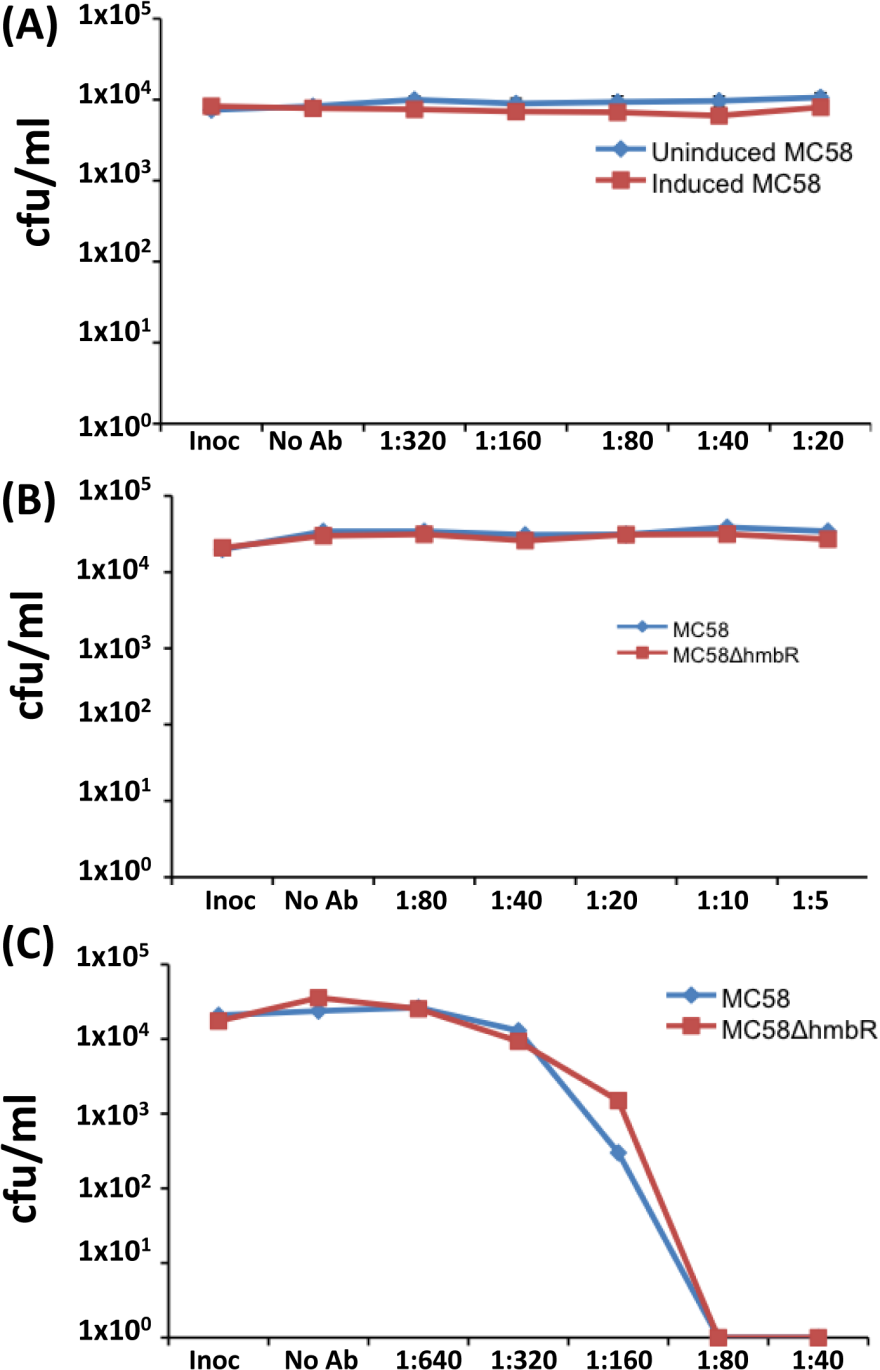
Testing the serum bactericidal assay of anti-HmbR antisera. Meningococcal cell suspensions, grown in iron-replete (uninduced) and iron-deficient (induced) conditions, and assays were performed as described for Fig 7. Antisera/antibodies were tested at the indicated dilutions for each sera against the indicated strains:- (A) polyclonal anti-rHmbR antisera, blue line, uninduced MC58, red line induced MC58; (B) pooled anti-HmbR mAbs, blue line induced MC58, red line induced MC58ΔHmbR; (C) a PorA monoclonal antibody, P1.7, blue line induced MC58, red line induced MC58ΔHmbR. Inoc, cell count after 0 minutes; No Ab, cell count after 60 minutes incubation in serum alone. Graphs show mean values for two independent experiments for the pooled anti-rHmbR antisera and one for the PorA P1.7 and anti-HmbR mAbs.

For the anti-HpuA hSBAs, a 5% human serum extract was used due to the high sensitivity of strain 8047 to human serum. The anti-r8047-HpuA serum exhibited no bactericidal activity against either strain 8047 *wt* and 8047Δ*hpuAB* (Fig 7A). Contrastingly, a P1.2 mAb specific for VR2 of PorA exhibited high levels of bactericidal activity for both strains with a titre of ≥ 640 (Fig 7B). The bactericidal activity of monoclonal and polyclonal anti-HmbR antibodies was performed using strain MC58, mutants thereof and 20% serum. Bactericidal activity was observed with an anti-PorA P1.7 mAb (hSBA titre of ≥ 320) but not with pooled anti-HmbR mAbs or anti-rMC58-HmbR antisera (Fig 8).

## Discussion

Hb is one of the most abundant sources of iron in the blood leading to the suggestion that haemoglobin utilisation is crucial for proliferation of blood-borne pathogens during systemic spread [10]. During meningococcal disease, Hb may be a particularly important iron source as the amount of iron-bound Tf molecules can be significantly reduced due to induction of a hypoferraimic response. Stojiljkovic *et al*. [29] showed that inactivation of *hmbR* in an *hpuAB*-negative meningococcal strain was incapable of Hb utilisation and was less virulent than the wild-type *hmbR*-positive strain in a rat infection model. Later genetic epidemiology studies revealed a bias towards the presence of *hmbR* and a high level of PV-ON states (70–95%) for one or both of the phase-variable Hb receptors in meningococcal disease isolates [27, 31, 32].

The exact nature of the involvement of the phase-variable Hb receptors in meningococcal virulence was examined using an *ex vivo* human whole blood model. Using a similar mutant to Stojiljkovic *et al*. [29], we observed that loss of the capacity to utilise Hb, via inactivation of *hmbR* in an *hpuAB*-negative strain (MC58), did not affect growth in human whole blood. Contrastingly, growth of a Δ*tbpBA* mutant was impaired in human whole blood. Our results support a critical role for TbpBA in infection as established by Zarantonelli *et al*. [50] who observed a drastic reduction in survival of a meningococcal *tbpB* mutant as compared to the parental strain during systemic infections of transgenic mice expressing human Tf. The disparity between our results and those of Stojiljkovic *et al*. [29] may be due to the meningococcal TbpBA preferentially binding human Tf [51, 52]. Thus the MC58∆*hmbR* mutant may not grow in the rat model due to an inability to utilise rat Tf but can grow in human whole blood by utilizing human Tf.

The lack of a role for HmbR during growth of meningococci in human blood is also surprising as Harrison *et al*. [31] detected a higher prevalence of the *hmbR* gene in meningococcal disease isolates than carriage isolates. The concentration of free Hb in blood is estimated at between 20–200 μg/ml depending on method of analysis and donor [53, 54]. Utilisation of free Hb by meningococci was tested with 100 μg in our assays, which is towards the upper bound of these estimates for free Hb in blood, and so it is possible that strain MC58 may not have grown in healthy human blood from these donors because the level of free Hb was too low. However, Zhao *et al*. [55] noted growth of wild-type meningococcal strains on 30–50 μg/ml free Hb and so it is not clear if the availability of free Hb was a determinant of the inability of *tbp* mutants to replicate in whole blood, The discrepancy may arise from the fact that the whole blood model only mimics the early stages of an infection where iron-loaded Tf is abundant [56, 57]. Thus the TbpBA receptor may be sufficient for growth of meningococci during the early stages of disease. However a decrease in Tf levels is anticipated over time during an infection and may occur concomitantly with an increase in intravascular coagulation and erythrocyte lysis [58, 59]. Lysis of the erythrocytes may release Hb in quantities that exceed the sequestration capacity (0.07–0.15 g/dL) of circulating Hp molecules [60] so that free Hb levels remain high. The potential importance of release of Hb was shown by Adamzik *et al*. who observed higher levels of free Hb in non-survivors as compared to survivors of sepsis [54]. Thus, an important role for HmbR may only become apparent following the removal of Tf and induction of erythrocyte lysis.

The presence of variable regions in the surface-exposed components of the Hb receptors indicates that immune responses against these receptors exert a strong selection for antigenic variation [28, 61]. Both receptors were immunogenic in mice and elicited antibodies that bound to surface-exposed regions of the proteins in whole cells as detected by FACs. These antibodies were not however bactericidal. The absence of bactericidal activity with the anti-HmbR mAbs was likely due to low levels of antibodies in the hybridoma supernatants whilst the inability of the anti-rHpuA and anti-rHmbR polyclonal antisera to mediate killing could be a consequence of the relatively low levels of the target surface-exposed epitopes. The importance of the amount of surface expression for discernible bactericidal activity was iterated by Giuntini *et al*. [62] who reported that from 4- to >10-fold higher amounts of anti-fHbp antibodies were required to elicit bacteriolysis of wild-type cells compared to a fHbp over-expression mutant. An inability of antibodies to mediate killing in an SBA, however, does not necessarily translate into an inability to provide protection against meningococci. West *et al*. [49] reported an incongruity between results obtained from SBAs and passive protection assays in mice. Antibodies produced in mice using an rTbpA immunogen were non-bactericidal in SBAs but the same rTbpA antigen, when used as a vaccine, protected mice from meningococcal infection. Against this backdrop, the anti-rHpuA and anti-rHmbR antisera may still limit meningococcal infection via promotion of opsonophagocytosis, which has been described for a non-bactericidal IgG3 antibody preparation induced in recipients of the Norwegian serogroup B meningococcal vaccine [63], or via the inhibition of Hb utilisation by preventing interactions between the Hb receptors and Hb or Hb complexes.

In summary, this study reports that the TbpBA receptor but not HmbR is required for meningococci to replicate in human blood. This finding suggests that TbpBA and HpuAB may be the main mediators of iron acquisition by meningococci during the early stages of an infection, due to the availability of apo-Tf and Hb-Hp complexes, whilst significant erythrocyte lysis may be required before HmbR contributes to the disease process. This study also finds that anti-rHpuA and anti-rHmbR antibodies are not bactericidal indicating that the meningococcal Hb receptors are not viable vaccine candidates.
